# Microvascular Architecture of Hepatic Metastases in a Mouse Model

**DOI:** 10.1155/1997/52739

**Published:** 1997

**Authors:** Darshini Kuruppu, Chris Christophi, Paul E. O'Brien

**Affiliations:** Department of Surgery Monash Medical School Alfred Hospital Prahran Victoria 3181 Australia

## Abstract

Development of effective treatment for hepatic metastases can be initiated by a better understanding of tumour vasculature and blood supply. This study was designed to characterise the microvascular architecture of hepatic metastases and observe the source of contributory blood supply from the host. Metastases were induced in mice by an intrasplenic injection of colon carcinoma cells (10^6^ cells/ml.) Vascularization of tumours was studied over a three week period by scanning electron microscopy of microvascular corrosion casts. Metastatic liver involvement was observed initially within a week post induction, as areas approximately 100 μm in diameter not perfused by the casting resin. On histology these spaces corresponded to tumour cell aggregates. The following weeks highlighted the angiogenesis phase of these tumours as they received a vascular supply from adjacent hepatic sinusoids. Direct sinusoidal supply of metastases was maintained throughout tumour growth. At the tumour periphery most sinusoids were compressed to form a sheath demarcating the tumour from the hepatic vasculature. No direct supply from the hepatic artery or the portal vein was observed. Dilated vessels termed vascular lakes dominated the complex microvascular architecture of the tumours, most tapering as they traversed towards the periphery. Four vascular branching patterns could be identified as true loops, bifurcations and trifurcations, spirals and capillary networks. The most significant observation in this study was the direct sinusoidal supply of metastases, together with the vascular lakes and the peripheral sinusoidal sheaths of the tumour microculature.


*HPB Surgery*, 1997, Vol. 10, pp. 149–158
Reprints available directly from the publisher
Photocopying permitted by license only

© 1997 OPA (Overseas Publishers Association)
Amsterdam B.V. Published in The Netherlands
by Harwood Academic Publishers
Printed in India

### Microvascular Architecture of Hepatic Metastases in a Mouse Model

DARSHINI KURUPPU, CHRIS CHRISTOPHI and PAUL E.O'BRIEN

Department of Surgery, Monash Medical School, Alfred Hospital, Prahran, Victoria 3181, Australia

*(Received 10 July 1995)*

Development of effective treatment for hepatic metastases can be initiated by a better understanding of tumour vasculature and blood supply. This study was designed to characterise the microvascular architecture of hepatic metastases and observe the source of contributory blood supply from the host. Metastases were induced in mice by an intrasplenic injection of colon carcinoma cells ($10^6$ cells/ml.) Vascularization of tumours was studied over a three week period by scanning electron microscopy of microvascular corrosion casts. Metastatic liver involvement was observed initially within a week post induction, as areas approximately 100 μm in diameter not perfused by the casting resin. On histology these spaces corresponded to tumour cell aggregates. The following weeks highlighted the angiogenesis phase of these tumours as they received a vascular supply from adjacent hepatic sinusoids. Direct sinusoidal supply of metastases was maintained throughout tumour growth. At the tumour periphery most sinusoids were compressed to form a sheath demarcating the tumour from the hepatic vasculature. No direct supply from the hepatic artery or the portal vein was observed. Dilated vessels termed vascular lakes dominated the complex microvascular architecture of the tumours, most tapering as they traversed towards the periphery. Four vascular branching patterns could be identified as true loops, bifurcations and trifurcations, spirals and capillary networks. The most significant observation in this study was the direct sinusoidal supply of metastases, together with the vascular lakes and the peripheral sinusoidal sheaths of the tumour microculature.

KEY WORDS: Intrasplenic model microvasculature corrosion casting blood supply liver metastases

### INTRODUCTION

Hepatic metastases accounts for over 70% of deaths from colorectal carcinoma. Apart from a sub group of patients who benefit from hepatic resection, other modes of therapy appear palliative. These include hepatic artery ligation or embolisation, selective internal radiation and regional chemotherapy. The majority of treatment modalities are based on the concept that the metastases receive a predominant hepatic arterial supply[1,2]. However, some tumours obtain a portal vein supply or establish arterioportal anastomoses[3–6] in order to compensate for arterial loss in treatment targeting the hepatic artery. These observations suggest that the supply of hepatic metastases is not exclusively arterial, and can be from sources other than the hepatic artery.

During initial growth a tumour survives on diffusion until it reaches 1–2 mm in diameter[7]. Further growth is dependant on angiogenesis, the process by which new capillary blood vessels are generated from preexisting capillaries and venules, induced by growth factors released by the tumour and from those present in the host milieu[8–10]. The vasculature of a mature tumour is composed of vessels derived from neovascularization, and modified preexisting host vessels[11,12]. The process of tumour angiogenesis is indefinite and is limited by eradication of the tumour or death of the host. Therefore, understanding the precise contribution of hepatic vessels in the establishment of liver metastases is crucial in the development of new treatment methods to curtail metastatic growth.

*Correspondence to*: Dr. C. Christophi, Department of Surgery, Monash Medical School, Alfred Hospital, Prahran, Victoria 3181, Australia. Phone: 61(03) 276 2608 Fax: 61(03) 510 3365

The aim of this study was to characterise the vascular architecture of experimental liver metastases over a period of growth, and to identify the source of contributory blood supply from the host.

### MATERIALS AND METHODS

### *Animals*

6–8 week old male inbred CBA mice were obtained from the Monash Univeristy Central Breeding Unit. They were fed on GR2 pellets and water ad libitum and housed under standard environmental conditions. Ethical approval for this study was granted by Monash University Animal Experimentation Ethics Committee and all animal experiments were conducted according to their guidelines.

### *Preparation of Cell Suspension For Induction*

The cell line CoMeMo was derived from a Dimethyl hydrazine (DMH) induced primary colon carcinoma in the mouse[13]. It was maintained *in vivo* by serial passage in the flank of CBA mice. To prepare the cell suspension, the mouse was killed and the flank tumour excised, minced, and dissociated by incubating with 0.1% trypsin in phosphate buffered saline (PBS) for 5 min. Trypsin was inactivated with complete Dulbecco's Modified Eagles Medium (DMEM) supplemented with 10% Foetal Calf Serum (FCS) (Flow Laboratories, Australia) and centrifuged for 5 min at 1000 rpm (Beckman Model TJ-6 Centrifuge, Australia). The pellet was resuspended in 0.9% saline. A viable cell count was done by trypan blue exclusion (0.05% trypan blue in distilled water), and the cell concentration was adjusted to $1 \times 10^6$ cells/ml.

### *Induction of Metastases*

The mouse was anaesthetised with an intraperitoneal injection of pentobarbitone sodium (Boehringer Ingelheim Pty Ltd, NSW, Australia), at 60 mg/kg. The spleen was carefully exteriorised by a subcostal incision of 0.5 cm across the skin and abdominal muscles parallel to the rib cage on the left side of the mouse. 0.1 ml of the tumour cell suspension was injected into the apex of the spleen with a 30 gauge needle over a period of 30 s. After allowing 1.5 min for the tumour cells to enter the portal circulation a splenectomy was performed using a cautery. Following splenectomy the wound was sutured with 5/0 coated vicryl. Following recovery the mice were monitored daily until used for the experiment.

### *Preparation of Samples*

Microvascular casts of the liver were prepared at days 7, 10, 13, 16, 19, or 22 and the microvascular architecture was studied by scanning electron microscopy. Another group of mice were killed at each of the above time points for histological assessment. A minimum of five mice were studied at each time point.

### *Techniques*

*(1) Corrosion casting* The technique of Skinner *et al.*[14] was modified for use in mice. The mouse was anaesthetised and the thoracic aorta was exposed from the left side by an incision between the third and fourth ribs. Exposure of the aorta from the thorax protected the vascular morphology of the liver from being damaged. A 22 gauge intravenous catheter was placed caudally into the aorta and positioned with ties. The vascular system was flushed with a saline solution infused via the catheter at a constant pressure of 120 mmHg at 37°C. The saline solution was composed of 0.154 M NaCl, with added polyvinylpyrrolidone 40, 60 mg/litre (Sigma, Australia), heparin, 10,000 units/litre (Weddel Pharmaceuticals, NSW, Australia), and papaverine 0.25 ml/litre (David Bull Laboratories, Victoria, Australia). An incision on the right artrium allowed free efflux of the washout. When the effluent was clear, an acrylic resin solution was infused via the same tubing at a constant pressure of 170 mmHg. The resin was prepared by mixing 5 ml of Mercox with 0.034 g of catalyst MA (Vilene Med. Co., Japan), and adding 1.67 ml of methyl methacrylate monomer (BDH Chemicals, Poole, UK) prior to infusion. When the resin flowed freely from the right atrium, the infusion cannula and the inferior vena cava were clamped and the resin was allowed to polymerise overnight at room temperature. The liver was carefully excised, digested in 20% KOH for 2 weeks, and washed in distilled water for a week. The microvascular cast was frozen and areas of interest were sectioned with a high speed saw (Dremel Moto-Tool, Model 396, USA) into approximately 1 mm sized cubes and air dried at room temperature for 2 days. Each piece was mounted on an aluminium stub (1 cm in diameter) with electrodag 415 (Acheson Colloids Company, MI, USA), ans coated with gold-palladium (96%:4%) in a sputter coater (Polaron sputter coater, Model E5000), and viewed on a SEM (Cambridge 250 Stereoscan).

*(2) Light microscopy* The mouse was killed with an over dose of anaesthetic and the liver was excised and fixed in 10% formalin in 100 mM phosphate buffer, pH 7.2 for a week and then stored in 50% ethanol. Approximately 2.0 mm thick slices from areas of interest were sectioned from the liver. They were dehydrated in a series of graded ethanol solutions, cleared in chloroform, paraffin embedded, and sectioned at 3 μm with a Spencer 820 microtome. The cut sections were stained with haematoxylin and eosin, mounted in D.P.X. (n,n dibotylphthalate polystyrene xylene) and examined under light microscopy (Olympus System Microscope, model BHT, Japan).

### *Calculation of Vascular Diameter from Casts*

The vascular diameter of tumour microvessels in corrosion casts was measured by scanning electron microscopy as indicated by the scales bar which appears at the foot of each micrograph.

### *Characterisation of Metastases*

The microvascular casts of normal and metastatic liver was observed by SEM and were characterised as follows: (i) Vascular distribution of normal liver (ii) morphological description of tumour microvasculature with growth, (iii) vascular branching patterns of tumours, and (iv) vascular supply of metastases from the host.

### *Statistical Analysis*

Data were expressed as the mean ± standard deviation. They were compared using a multiple analysis of variance, and by post hoc comparison using a least significant difference test (Statsplus, StatsSoft, Okalahoma, USA). P values less than 0.05 were considered significant.

### RESULTS

### *i) Normal Liver Microvasculature*

The spatial arrangement of the normal hepatic vessels was clearly elucidated in the microvascular casts. All vessels were patent as observed by complete filling by the resin. On cross section the hepatic lobules were outlined by the portal tracts. The portal veins tapered into sinusoids which drained into the central vein at right angles (Fig. 1a). Branches of the hepatic artery supplied the peribiliary plexus. Bile ducts and lymphatic vessels are not identified by this technique. No arterio-portal anastomoses were seen. The mouse liver sinusoids had a mean diameter of 6.8 ± 1.7 μm. Except for an occasional terminal portal vein only sinusoids were seen on the surface of the liver (Fig. 1b).

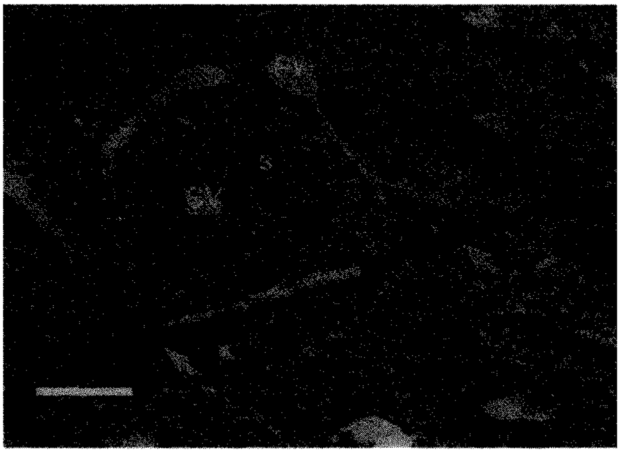

**Figure 1a** A microvascular cast of a normal mouse liver sectioned across two lobes, the arrows indicating the space between the lobes. All vessels are filled completely by the resin. Portal veins (PV) outline hepatic lobules. Sinusoids(S) coalesce towards the central vein (CV). Mouse sinusoids have a diameter of 6.8 ± 1.7 μm. Bar=200 μm.

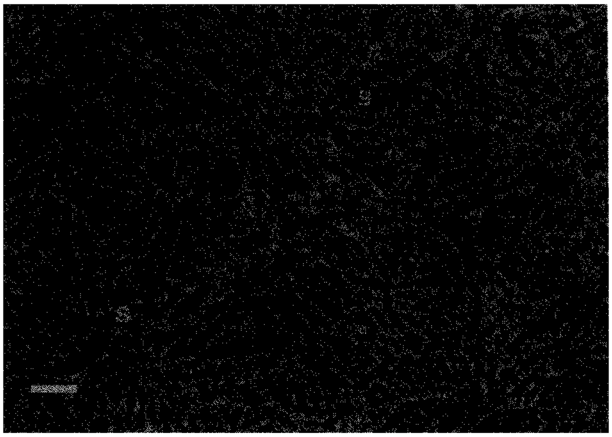

**Figure 1b** The surface of a microvascular cast of a normal mouse liver. Sinusoids (S) traverse randomly with no specific pattern of distribution. Bar=100 μm.

### *(ii) Morphological Characteristics of Metastases*

Hepatic involvement by malignant disease was first evident in microvascular casts one week post tumour induction. Circular areas with a mean diameter of 145.0 ± 50.7 μm were observed within the sinusoidal network which represented regions left unperfused by the casting resin (Fig. 2a). These areas were distributed closer to the central vein and were outlined by blind ended sinusoids. On histology these spaces corresponded to tumour cell aggregates (Fig. 2b). By day ten these nonpatent areas had reached a mean diameter of 318.5±317:1 μm. Vascularised tumours with a mean diameter of 0.4±0.1 mm were present by this time, which were visible macroscopically. Tumour microvasculature increased in complexity with growth deviating considerably from the standard organisation of the normal hepatic vasculature. Due to abnormal diliations and tortuosity, these vessels were described as vascular lakes. The lakes observed initially with tumour vascularisation communicated with the liver sinusoids at the tumour-host interface (Fig. 3). The sinusoidal continuity of the tumour vessels was maintained throughout tumour growth, increasing in sites of communication. By 3 weeks tumours had reached a diameter 3.0±1.1 mm and had almost completely replaced the hepatic vasculature.

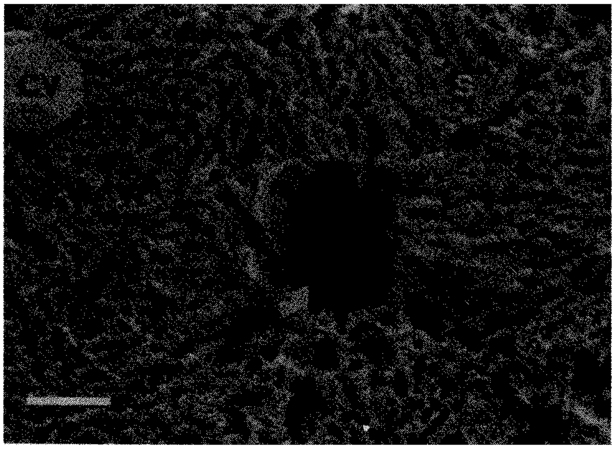

**Figure 2a** A microvascular cast of a liver one week post induction of metastases depicting initial signs of hepatic infiltration by tumor. Areas approximately 100 μm in diameter (arrow) within the sinusoidal network (S) are left unfilled by the casting resin. These regions are situated closer to the central vein (CV). Bar=100 μm.

The vascular lakes were lined by an endothelial monolayer. They were distributed in the tumour centre and tapered towards the periphery as they communicated with the liver sinusoids. Vascular dimensions at the beginning of tumour vascularization (day ten) were 42.9±7.0 μm and 13.1±6.0 μm in central and peripheral regions of the tumour. By three weeks the respective diameters reached 216.0±35.8 μm and 60.8±20.3 μm (Table 1). Hepatic sinusoids adjacent to the tumour remained distended

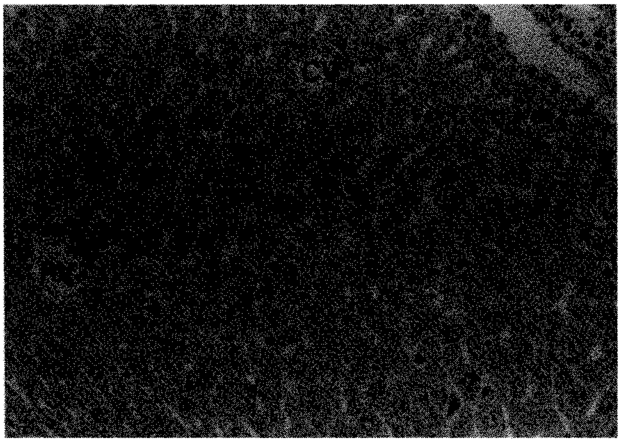

**Figure 2b** A Hand E stained mouse liver at 220×magnification. Tumor cells (arrow) observed in the sinusoids (S) are closer to the central vein (CV). These tumor cell aggregates may correspond with the nonpatent areas observed in casts (Fig. 2a). PV=portal vein.

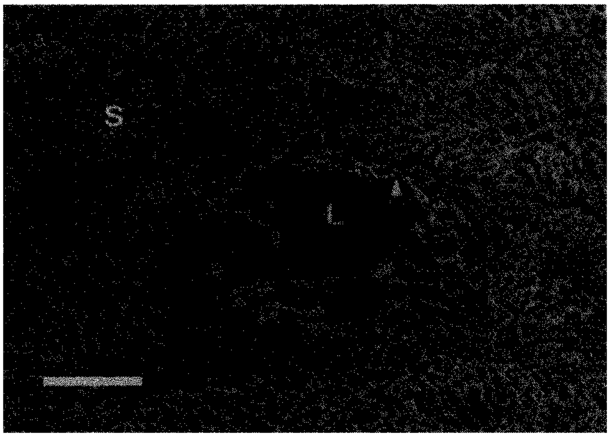

**Figure 3** A microvascular cast of a metastatic liver with early evidence of tumor angiogenesis. Large tumor vessels termed vascular lakes (L) maintain continuity with the liver sinusoids (S) at the tumor periphery (arrow head). This communication was maintained throughout the further growth phase. Bar=200 μm.

throughout tumour growth, while those distant to the tumours seemed to be affected only by the third week, increasing their diameter to 12.3±2.6μm ($p$=0.005) (Table 1).

With tumour growth the hepatic sinusoids adjacent to the metastases were compressed to form a sheath encircling them. Small tumours were surrounded by 2–3 compressed sinusoids (Fig. 4) Larger tumours were encapsulated by a thick band of compressed sinusoids (approximately 130 μm in diameter) demarcating the tumour from the normal liver vasculature (Fig. 5a and 5b). Sinusoidal continuity of the tumour vascular lakes were still maintained by the innermost layer of the sinusoidal sheath.

**Table 1** Variation in size of tumor vessels and hepatic sinusoids with tumor growth.

| | *Tumor Vessels* | | *Hepatic Sinusoids* | | |
|---|---|---|---|---|---|
| *Days Pos: Induction* | *Tumor Centre* | *Tumor Periphery* | *Adjacent to Tumor* | *Distant to Tumor* | *n* - |
| 10 | 42.9±7.0 | 13.1±6.0 | 21.0±7.2 | 6.4±1.1 | 7 |
| 13 | 125.4±61.5* | 46.3±16.5* | 28.6±19.1 | 7.2±1.5 | 16 |
| 16 | 128.6±22.7 | 56.1±25.4 | 31.8±14.5 | 8.8±1.8 | 14 |
| 19 | 177.5±54.0 | 54.6±16.8 | 26.6±7.5 | 9.4±2.1 | 15 |
| 22 | 216.0±35.8 | 60.8±20.3 | 27.9±6.6 | 12.3±2.5** | 18 |

Data are expressed as the mean ±s.d. of vessel diameter (μm)
*p<0.002 compared to day 10.**p=0.005 compared to day 19

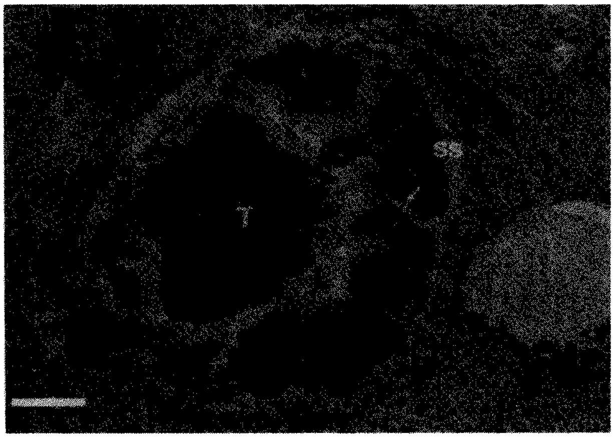

**Figure 4** A cross section of a tumor at 2 weeks post metastases induction. Compressed sinusoids form a sheath (SS) encircling the tumor (T). Tumor vascular lakes (L) still maintain continuity with the hepatic sinusoids (S) via this sheath (arrow). Bar=200 μm.

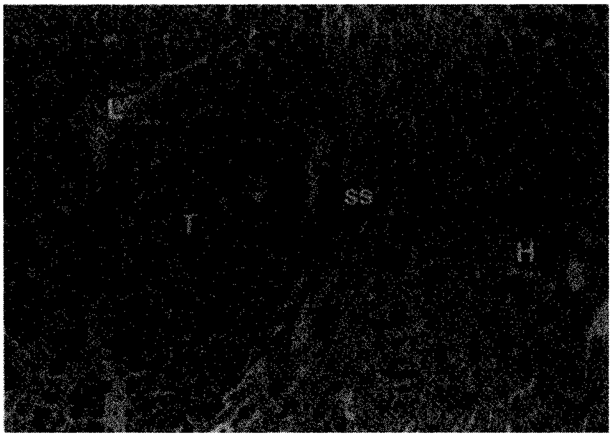

**Figure 5b** A Hand E section of a metastatic liver at 3 weeks post induction. The tumor (T) is demarcated from the hepatic tissue (H) by a band of compressed hepatic sinusoids (SS) which correspond with the sinusoidal sheath observed in casts (Fig. 5a). L=tumour vascular lakes. Magnification 220×.

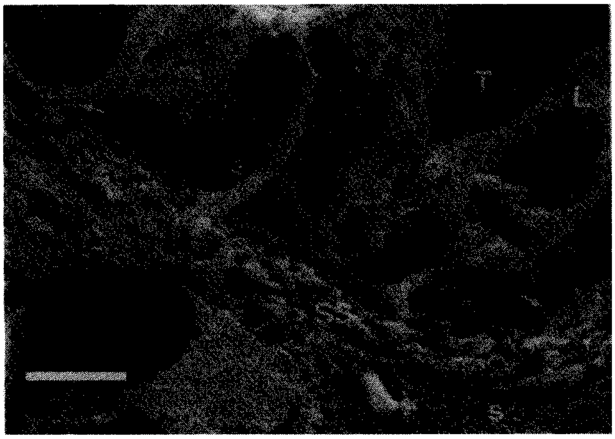

**Figure 5a** A cross section of a metastatic liver cast at 3 weeks post metastases induction. The sinusoidal sheath (SS) is thicker than at 2 weeks (Fig. 4), and demarcate the tumor (T) from the hepatic vasculature (S). Sinusoidal continuity of the tumor lakes (L) is maintained via the sheath (arrows). Bar=200 μm.

*(iii) Vascular Branching Patterns of Tumours*

Four branching patterns were observed in the vasculature of metastases. They are: true loops, spirals bifurcations and trifurcations and capillary networks. "Spirals" is a descriptive term given to a branching pattern seen in the metastases in this study.

*(1) True loops* A true loop consists of vessel segments with side branches, distributed in different planes (8). True loops were observed frequently in the central region of the metastases and areas immediately adjacent. Their diameter varied between 25–50 μm (Fig. 6a).

*(2) Spirals* A medium sized vessel (40–50 μm in diameter) branching in alternate planes around a central axis was termed a spiral. These branches tapered and decreased in length as they reached the free end.

Spirals were observed in the centre of the metastases (Fig. 6b).

*(3) Bifurcations and trifurcations* These were the most common branching patterns in the mouse metastases. They were seen frequently in all regions of the tumors and consisted of vessels of varying diameter

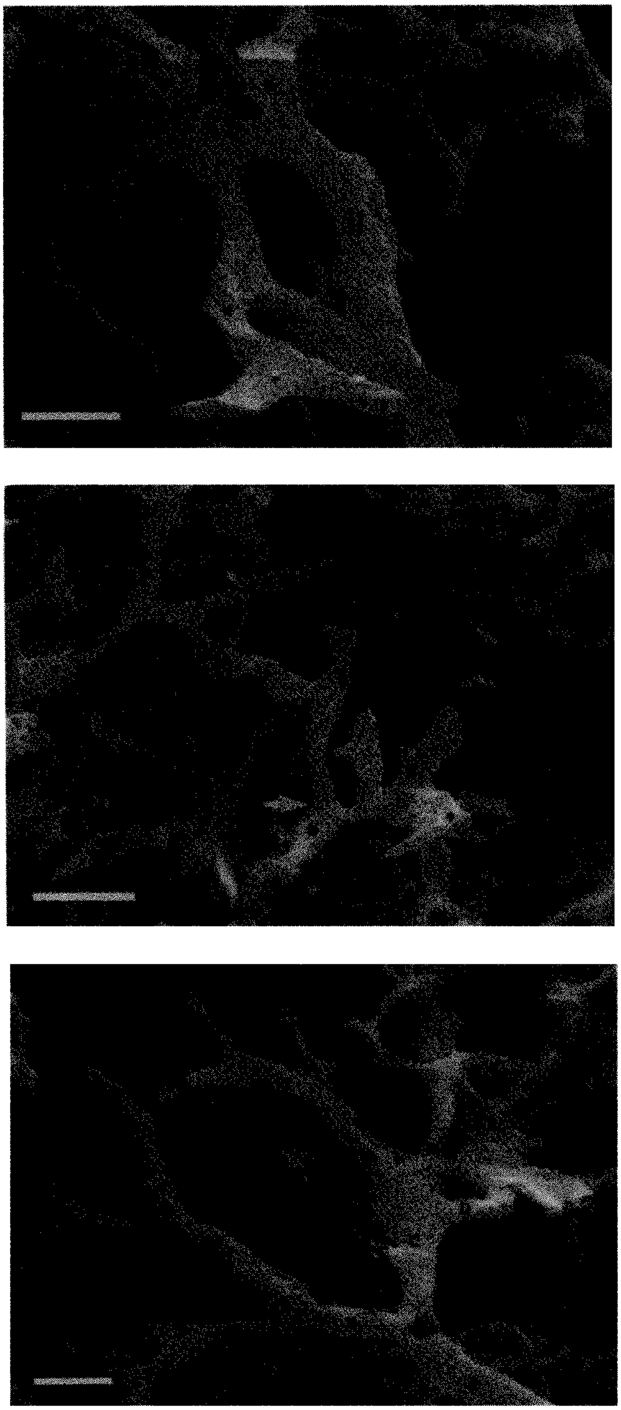

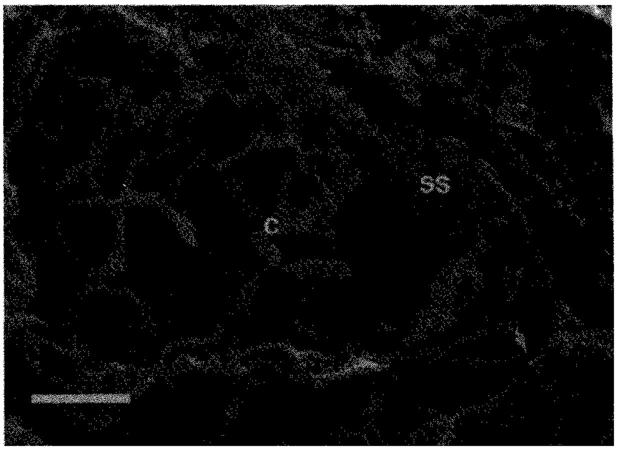

**Figure 6** Vascular branching patterns of tumors. (1) True loop:- the true loop (tl) has vessel segments (asterisks) which are on different planes to the remainder of the loop. Bar=100 µm. (2) Spirals:-branches (b) arises from the base of a vessel (arrow) in alternate planes and taper and decrease in length as they reach the free end (asterisk). Bar=200 µm. (3) Bifurcations and Trifurcations:- the large vessel (A) bifurcates (B). The right branch again bifurcates (C). of which one is broken. The other trifurcates immediately (D). The diameter of the branches decrease in diameter from 50 µm in branch (A) to half the size in branch (D). Bar=100 µm. (4) Capillary network:- the capillary network (C) is composed of vessels of similar diameter which communicate (arrow) with the sinusoidal sheath (SS). The liver sinusoids (S) are seen beyond the sheath. Bar=400µm.

(Fig. 6c). Most often vascular lakes either bifurcated or trifurcated decreasing in diameter.

*(4) Capillary networks* A capillary network was composed of tapering branches of the vascular lakes. They were 6–7 µm in diameter and were randomly distributed at the tumour periphery where a few of the branches communicated with the sinusoidal sheath (Fig. 6D).

### *(iv) Vascular Supply of Metastases from Host Origin*

All microvascular casts studied showed strong evidence that liver metastases in this model were supplied by hepatic sinusoids. Hepatic sinusoidal contribution was seen initially with tumour vascularization at day ten (Fig. 3). Sinusoidal continuity with the tumour microvascular lakes was maintained throughout tumour growth, increasing in sites of communication at the tumour-host interface (Fig. 7). Dimensions of the hepatic sinusoids were altered as they traversed towards the tumour vasculature, most often dilating adjacent to the tumour periphery (Table 1). The compressed sinusoids adjacent to the tumour periphery formed a sheath encircling the tumour. Although small metastases communicated directly with the liver sinusoids, most larger lesions communicated with the sinusoids via the sinusoidal sheath (Fig. 4). Direct communications between tumour vessels and hepatic arteries or the portal veins was not observed in any of the 51 tumours studied. However, occasional communications with the central vein were seen.

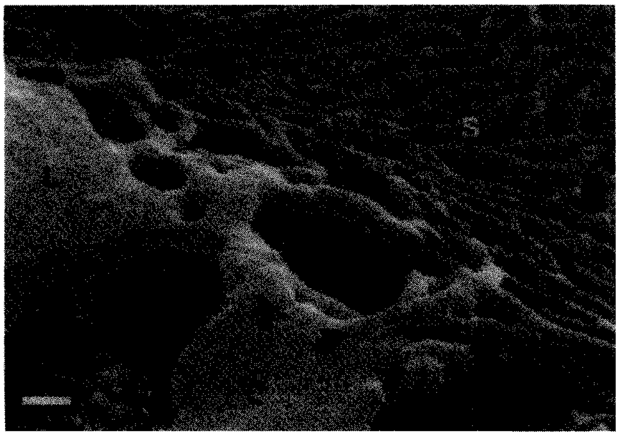

**Figure 7** Sinusoidal supply of metastases. Tumor vascular lakes (L) communicate with the hepatic sinusoids (S) via branches travelling towards the host vasculature (arrows). Sinusoidal supply of metastases was evident throughout tumor growth. However, no arterial or portal supply was observed. Bar=40 μm.

### DISCUSSION

Controversy surrounding the precise contribution of host vessels in nourishing hepatic metastases has argued the validity of current treatment modalities. The present study provides clear evidence that liver metastases are supplied by the hepatic sinusoids. Sinusoidal supply which was observed from the time of tumour vascularization was maintained throughout tumour growth. This study challenges the concept that liver metastases receive an exclusive arterial supply, and adds an insight as to why treatment targeting the hepatic artery fails subsequently.

Metastatic liver involvement was first evident in the present model in microvascular casts as non perfused circular regions, which correspond to tumour cell aggregates on histology. Studying microfil perfused tumour implants in the liver by stereomicroscopy, Ackerman reported "blank spaces" which corresponded to tumour implants less than 1 mm[15]. MacPhee *et al.* reported spherical avascular regions with a mean diameter of $83 \pm 26$ μm to be characteristic of hepatitis-infected mouse livers[16]. Although the hepatic artery is known to supply liver metastases[6,17], recent studies indicate a portal contribution[15,18]. In the present study no direct arterial or portal supply of metastases was seen. The metastases however received a dominant sinusoidal supply which was maintained throughout tumour growth. Vessels of small tumours communicated directly with the liver sinusoids at the tumour-host interface. As the tumour mass increased the sinusoids adjacent to the tumour were compressed to form a sheath encircling them. Communication of the tumor vessels in large lesions with the liver sinusoids was further maintained via the sinusoidal sheath. Compressed sinusoids close to tumours have also been observed by *in vivo* microscopy of liver tumours[19]. Communication of tumour vessels with hepatic sinusoids have been observed in human hepatocellular carcinoma by SEM of vascular casts[20,21]. A study observing metastases of varying primaries at autopsy by gelatine perfusion and resin corrosion techniques reveal that metastases up to 200 μm receive their main blood supply from the sinusoids[22]. Some studies have reported an occasional communication of tumour vessels with the surrounding liver sinusoids[17,19].

A few tumours in our model also showed communications of the tumour vascular lakes with the central vein. Hepatic metastases have been shown to be supplied by portal venules and drained by hepatic venules[19,22]. According to observations from the present study metastases may receive a sinusoidal supply and drain into the central vein, or may indicate a reverse flow to the sinusoids.

Another interesting observation in our study was the development of dilated vascular lakes within the central and peripheral regions of tumours. The vessels tapered as they reached the periphery to communicate with the hepatic sinusoids. More interesting was the gradual increase in vessel diameter with increase in tumour mass (Table 1), suggesting a continuous strengthening of the vascular architecture of the metastases with growth. Dilated vessels seen in our study are characteristic of metastases from colon cancer in experimental models[6,13,17] and in humans[18,22]. The blood supply in these vascular lakes diminished with metastatic growth as observed by laser doppler flowmetry in this model[13]. The dilated tumour vascular lakes would explain the sluggish blood flow of the metastases.

The present study is the first to describe branching patterns of liver metastases, and we report four different vascular branching patterns: true loop, bifurcations and trifurcation, capillary networks and spirals. Except for the latter branching pattern, others were named according to the classification scheme of Less, Skalak and Jain[8] which have been described for mammary carcinoma.

The normal hepatic vasculature was replaced almost completely by the tumour vasculature by three weeks. This observation is further strengthened by stereological analysis of metastases of this model which revealed that with initial growth at day ten metastases occupied 2.19 % of the liver and gradually occupied 59.15 % of the liver by 3 weeks[23]. Tumours in the present study were located predominantly in the centrilobular region. This is in contrast of observations which support the arrest of tumour cells in the periportal sinusoids[24]. Features which promote a periportal arrest include a higher proportion of anastomoses, diminished pressure and flow, greater number of Kupffer cells, and endothelial cells with receptor ligand properties[25]. Decreased expression of the above features may predispose lodgement of cells in the centrilobular region. Colon carcinoma cells used in our model seem to have bypassed entrapment and destruction in the periportal and intermediate zones. This cell line may also express ligands for cellular receptors in the centrilobular region.

Most *in vivo* and *in vitro* techniques employed to study the morphology of the tumour microvasculature include transparent chambers[11,26], angiography[5,19,24], and infusion studies[17,20]. Although transparent chambers allow direct visualisation of angiogenesis *in vivo*, the natural expansion of the vasculature is constrained to the chamber area. Arteriographic studies limit the microcirculation to two dimensional visualisations. Resolution of infusion techniques is regulated by the injection material and viewing technique. SEM of microvascular corrosion casts, although an indirect and a non dynamic technique, offers three dimensional representations of the microvascular architecture. It has been used successfully to study the morphology of liver[27,28], and liver tumours[29,30] in experimental animals and in humans. High spatial resolution and depth of focus elucidates intricate details of vascular beds, which were hitherto obscure by conventional methods. The mouse metastases, observed by scanning electron microscopy, allowed visualisation of the smallest tumour vessels.

Extravasation of casting material was noted in several tumour vessels. This could represent increased permeability, rarefaction, haemorrhage, or necrosis of tumour vessels. Tumour vessels are known to be more permeable than normal tissue[31]. Ausprunk and Folkman[12] have shown that during the formation of capillary sprouts endothelial cell migration resulted in loosening of cell junctions and dissolution of the basal lamina creating gaps in the vascular wall through which erythrocytes extravasated. Extravasation of casting material from tumour vessels can therefore indicate angiogenesis activity. Skinner *et al.*[14], studying the vascularisation of colon tumours by corrosion casting have described extravasation of casting material from tumour vessels as possible sites of angiogenesis. Sites of extravasation in this system can be clearly detected and does not interfere with observing the microvasculature in casts. The casting saline and resin employed together with the infusion pressues selected in this study allow complete filling of the microvessels without distorting them or creating hindering artifacts.

Tumour cell proliferation is dependant on the concurrent growth of a supportive vasculature initiated by tumour released substances or host factors triggered by the tumour[7,9,32]. Tumour cell aggregates observed in the microvascular casts at day 7 may have initiated an angiogenic response from nearby sinusoids. This can be suggested as slight extravasation of casting material was also observed in a few sinusoids immediately adjacent to the tumour. As areas of vascular proliferation are prone to haemorrhage and can be observed as sites of extravasation of casting resin, extravasation seen in the hepatic sinusoids may indicate the contribution of these vessels in the vascularisation of tumours. Therefore, tumour vasculature may be a result of angiogenesis from existing host vessels (sinusoids) and modified host sinusoids.

This is the first study to provide strong evidence that hepatic metastases are supplied by the liver sinusoids. Studies which indicate similar observations are few and inconclusive. Sinusoidal supply of metastases indicates that the tumours receive both portal and arterial blood. A substantial supply of metastases from the sinusoids may explain unsatisfactory outcome of treatment targeting the hepatic artery or the portal vein. If dominant sinusoidal supply of tumours is present, new avenues for implementing treatment strategies may become possible.

### REFERENCES

1. Breedis, C. and Young, G. (1954) The blood supply of neoplasms in the liver. *Am. J. Path.*, **30**, 967–977.

2. Healey, J.H. (1965) Vascular patterns in human metastatic liver tumours. *Surg., Gynaecology and Obstetrics*, **120**, 1187–1193.
3. Taylor, I., Bennett, R. and Sherriff, S. (1979) The blood supply of colorectal liver metastases. *Br. J. Cancer*, **39**, 749–755.
4. Goldberg, J.A., Thomson, J.A.K., McCurrach, G., Anderson, J.H., Wilmott, N., Bessent, R.G., McKillop, J.H. and McArdle, C.S. (1991) Arteriovenous shunting in patients with colorectal liver metastases. *Br. J. Cancer*, **63**, 466–468.
5. Carlsson, G., Ekelund, L., Stigsson, L. and Hafstrom, L. (1983) Vascularization and tumour volume estimations of solitary liver tumours in rats. *Annales Chirgiae et Gynaecologiae*, **72**, 187–191.
6. Cagol, P.P., DaPian, P.P., Kiotto, D., Pilati, P.L., Pasqual, E.M., Talenti, E. and Lise, M. (1989) Vascularization of experimental liver metastases. 1-Blood supply and vascular patterns. *J. Exp. Clin. Cancer Res.*, **8**, 113–122.
7. Folkman. J. (1985) Tumour angiogenesis. *Adv. Cancer Res.*, **43**, 175–203.
8. Less, J.R., Skalak, T.C., Sevick, E.M. and Jain, R.K. (1991) Microvascular architecture in a mammary carcinoma: branching patterns and vessel dimensions. *Cancer Res.*, **51**, 265–273.
9. Warren, B.A. (1979) The vascular morphology of tumours. In: Tumour blood circulation: Angiogenesis, Vascular Morphology and Blood Flow of Experimental and Human Tumours, edited by HI Peterson, *FL: CRC Press Inc*, **1979**, 1–47.
10. Jain, R.K. (1988) Determinants of tumour blood flow: a review. *Cencer Res.*, **48**, 2641–2658.
11. Eddy, H.A. and Casarett, G.W. (1973) Development of the vascular system in the hamster malignant neurilemma. *Microvasc. Res.*, **6**, 63–82.
12. Ausprunk, D.H. and Folkman, J. (1977) Migration and proliferation of endothelial cells in preformed and newly formed blood vessels during tumor angiogenesis. *Microvasc. Res.*, **14**, 53–65.
13. Kuruppu, D., Christophi, C., Bertram, J. and O'Brien, P.E. (1996) Characterisation of an animal model of hepatic metastases. *J. Gastroenterology and Hepatology*; In Press.
14. Skinner, S., Tutton, P.J.M. and O'Brien, P.E. (1990) Microvascular architecture of experimental colon tumours in the rat. *Cancer Res.*, **50**, 2411–2417.
15. Ackerman, N.B. (1974) The blood supply of experimental liver metastases. IV. Changes in vascularity with increasing tumour growth. *Surgery*, **75**, 589–596.
16. MacPhee, P.J., Schmidt, E.E., Keown, P.A. and Groom, A.C. (1988) Microcirculatory changes in livers of mice infected with murine hepatitis virus. Evidence from microcorrosion casts and measurements of red cell velocity. *Microvascular Res.*, **36**, 140–149.
17. Ekelund, L. Lin, G. and Jeppsson, B. (1984) Blood supply of experimental liver tumours after intraarterial embolisation with gelfoam powder and absolute ethanol. *Cardiovas. Intervent. Radiol.*, **7**, 234–239.
18. Lin, G., Lunderquist, A., Hagerstrand, I. and Boijsen, E. (1984) Postmortem examination of the blood supply and vascular pattern of small liver metastases in man. *Surgery*, **96**, 517–526.
19. Kan, Z., Ivancev, K., Lunderquist, A., McCuskey, P.A., Wright, K.C., Wallace, S. and McCuskey, R.S. (1993) *In vivo* microscopy of hepatic tumours in animal models: a dynamic investigation of blood supply to hepatic metastases. *Radiology*, **187**, 621–626.
20. Kita, K., Itoshima, T. and Tsuji, T. (1991) Observation of microvascular casts of human hepatocellular carcinoma by scanning electron microscopy. *Gastroenterologia Japonica*, **26**, 319–328.
21. Sugihara, S., Kogiro, M. and Nakashima, T. (1985) Ultrastructural study of hepatocellular carcinoma with replacing growth pattern. *Acta Pathol. Jpn.*, **35**, 549–559.
22. Haugeberg, G., Strohmeyer, T., Lierse, W. and Bocker, W. (1988) The vascularization of liver metastases: histological investigation of gelatine injected specimens with special regard to the vascularization of micrometastases. *J. Cancer Res. Clin. Oncol.*, **114**, 415–419.
23. Egawa, J., Ishioka, K. and Ogata, T. (1979) Vascular structure of experimental tumours: appearances in scanning electron microscope. *Acta Radiologica Oncology*, **18**, 367–375.
24. Barbera-Guillem, E. and Vidal-Vanacloca, F. (1991) Selective involvement of a specific sinusoidal domain in hepatic metastasis. The Microcirculation of Tumours, edited by FW Orr, MR Buchanan and LFL Weiss: CRC Press inc, 183–203.
25. Barbera-Guillem, E., Alonso-Varona, A. and Vidal-Vanacloca, F. (1989) Selective implantation and growth in rats and mice of experimental metastases in acinar zone one. *Cancer. Res.*, **49**, 4003–4010.
26. Endrich, B. and Messmer, K. (1981) Microcirculation of transplanted tumours. *Arzneim-Forsch.*, **31**, 2007–2011.
27. Ohtani, O., Kikuta, A., Ohtsuka, A., Taguchi, T. and Murakami, T. (1983) Microvasculature as studied by the microvascular corrosion casting/scanning electron microscope method. I. Endocrine and digestive system. *Arch. Histol. Jap.*, **46**, 1–42.
28. Grisham, J.W. and Nopanitaya, W. (1981) Scanning electron microscopy of casts of hepatic microvessels: review of method and results. In: Hepatic Circulation if Health & Disease, edited by WW Laut. New York: Raven, 87–109.
29. Tsuda, H., Tamano, S., Imaida, K., Ohshima, M., Kitahori, Y., and Ito, N. (1984) Three dimensional observations by scanning electron microscopy on the blood supply and organization of vasculature during hepatocarcinogenesis in rats. *Acta Pathol. Jpn.*, **34**, 957–970.
30. Yamamoto, K., Sherman, I., Phillips, M.J. and Fisher, M.M. (1985) Three dimensional observations of the rat hamster and human liver by scanning electron microscopy of microvascular casts. *Hapatology*, **5**, 452–456.
31. Ackerman, N.B. and Hechmer, P.A. (1978) Studies on the capillary permeability of experimental liver metastases. *Surgery, Gynecology & Obstetrics*, **146**, 884–888.
32. Shubik, P. (1982) Vascularisation of tumours: A review. *J. Cancer Clin. Oncol.*, **103**, 211–226.

### COMMENTARY

The vascular network within and surrounding tumour tissue will have a major impact upon the micro environment and will not only influence growth and spread but is important for drug delivery and other therapeutic measures. In this study the authors have used a very elaborate technique of studying microvascular architecture of hepatic metastases in a mouse model. They have in an elegant way characterised the surrounding of small liver metastases and made the observation that there is a direct sinusoidal supply of metastases as well as vascular lakes and peripheral sinusoidal sheath of tumour microvasculature. This finding that established liver tumours have supply not only from the arterial side may explain some of the failures of therapies directed at interrupting the arterial bloodflow or use it for delivery of tumoricidal agents. The information may also have some influence on the development of new drugs influencing tumour blood flow and angiogenesis[1].

This study is descriptive in nature and I hope that the authors continue on this line and try to add a more dynamic dimension of the matter to delineate the functional importance of the different vascular sources.

### REFERENCE

1. Tanaka, T., Konno, H., Matsuda I., Nakamura, S. and Baba, S. (1995). Prevention of hepatic metastasis of human colon cancer by angiogenesis inhibitor TNP-470[1]. *Cancer Research*, **55**, 836–839.

Bengt Jeppsson
Department of Surgery
Lund University Hospital
S-221 85 Lund
Sweden

### COMMENTARY

Colorectal liver metastases carry a poor prognosis. Only a very small proportion of patients are suitable for resection and the remaining are notoriously unresponsive to a range of treatments. More effective chemotherapy may be initiated by a better understanding of the tumour blood supply.

This paper investigates the blood supply of liver metastases in an animal model, by scanning electron microscopy of vascular corrosion casts. The results are interesting; small tumour deposits (upto 100 μm) do not have a direct blood supply and most probably receive nutrients by diffusion as previously described. Angiogenesis was visible when the tumour foci started growing; vascular supply was directly from the sinusoids which suggested that the metastases received both hepatic arterial and portal venous blood. No direct link was observed with the hepatic artery or portal vein. This observation does not correlate with the concept that most blood supply to liver metastases is from the hepatic artery. Further interesting observations included the presence of "vascular lakes" within the tumour. A technical point which should be taken into consideration is the physical effect of corrosion casting on blood vessels. The application of such techniques will trace the anatomy of all blood vessels irrespective of their *in vivo* status (ie, whether they are closed or open). Therefore anatomical results do not always correlate with physiological function.

This well presented study challenges the current belief on metastatic blood supply. However, while these findings are of potential importance, caution needs to be exercised when extrapolating them to the human situation where slightly different interactions may occur. Human results are eagerly awaited.

M. Loizidou and I. Taylor,
University College London Medical School
Department of Surgery,
The Institute of Surgical Studies,
Charles Bell House,
67-73 Pinding House Street,
London WIP 7LD

